# Translation and cross-cultural adaptation into Brazilian Portuguese of the ORAAprof instrument for Advanced Access

**DOI:** 10.1590/0034-7167-2024-0329

**Published:** 2025-12-08

**Authors:** Alexandre Ramiro Pinto, Lislaine Aparecida Fracolli, Lucia Yasuko Izumi Nichiata

**Affiliations:** I Universidade de São Paulo. São Paulo. São Paulo. Brazil

**Keywords:** Translating, Methods, Appointments and Schedules, Health Services Accessibility, Primary Health Care., Traducción, Métodos, Citas y Horarios, Accesibilidad a los Servicios de Salud, Atención Primaria de Salud.

## Abstract

**Objectives:**

to translate and cross-culturally adapt the self-reflective tool *Outil Réflexif sur l’Accès Adapté* (ORAAprof) into Brazilian Portuguese.

**Methods:**

methodological study conducted from August 2022 to April 2023 in five stages: translation, back-translation, synthesis of translations, evaluation by an expert committee, and authors’ approval.

**Results:**

independent specialists carried out the translations and back-translations. The Brazilian researchers produced the synthesis version based on those translations and back-translations. An expert committee evaluated the synthesis version considering linguistic aspects (semantic, idiomatic, cultural, and conceptual) and judged the final Portuguese version to be adequate; the Content Validity Ratio was 1.0. The final consensus version of the *Ferramenta Autorreflexiva sobre o Acesso Avançado para Profissionais* (ORAAprof Brasil) was approved by the original authors.

**Final Considerations:**

ORAAprof-Brasil may provide information to improve access quality and management in Primary Health Care.

## RESUMEN

**Objetivos:**

traducir y adaptar transculturalmente la herramienta autorreflexiva *Outil Réflexif sur l’Accès Adapté* (ORAAprof) al portugués brasileño.

**Métodos:**

estudio metodológico desarrollado de agosto de 2022 a abril de 2023 en cinco etapas: traducción, retrotraducción, síntesis, evaluación por un comité de expertos y aprobación de los autores.

**Resultados:**

las etapas de traducción y retrotraducción fueron realizadas por especialistas independientes. La versión de síntesis, basada en dichas versiones, la elaboró el equipo brasileño. El comité de expertos evaluó la versión de síntesis según la equivalencia lingüística (semántica, idiomática, cultural y conceptual) y la consideró adecuada en portugués, con Razón de Validez de Contenido a nivel de escala igual a 1,0. La versión consensuada *Ferramenta Autorreflexiva sobre o Acesso Avançado para Profissionais (ORAAprof-Brasil)* fue aprobada por los autores originales.

**Consideraciones Finales:**

ORAAprof-Brasil puede aportar información para mejorar la calidad y la gestión del acceso en la Atención Primaria de Salud.

## INTRODUCTION

According to the World Health Organization, the quality of healthcare services improves desired health outcomes. To achieve this, services are expected to deliver evidence-based care (effective), avoid harm (safe), and provide care that meets service users’ needs (person-centered). Thus, a crucial aspect is timely access, with a focus on reducing waiting times and delays^(1)^. Timely access also refers to a health service’s ability to schedule an appointment as quickly as possible in response to a service user’s need. Organized scheduling is argued to reduce costs and overcrowding at other levels of care^(2)^.

Timely access to health services is one of the greatest challenges facing health systems internationally^(3)^ and is often regarded as the primary barrier encountered by service users^(4)^. In Brazil, additional obstacles further hinder access to the Unified Health System (SUS), including underfunding, a high number of users per Family Health Strategy (ESF) team, shortages and uneven distribution of physicians, operational problems and undersized services, as well as excessive formality in appointment scheduling (token distribution and restricted time slots) and selective prioritization of groups^(5,6)^.

Improving access requires organizational strategies that balance supply and demand and promote integration^(7)^, such as knowing the size of the registered population (panel size) and the number of available appointments (schedule); rejecting the classification of “urgent” versus “non urgent”; and identifying the drivers of appointment demand^(8)^. Several appointment-scheduling models have been implemented to achieve this balance, including Open Access, Book-on-the-Day, Supersaturate, Carve-out, and Advanced Access^(9)^.

Advanced Access (AA) was developed in 1998 in the United States by physician Mark Murray and nurse Catherine Tantau^(10)^, and later spread to North America and Europe^(11,12)^. Under AA, a service user should be seen within 48 hours, with no formal distinction between urgent (walk-in) and routine (scheduled) visits. The slogan of this scheduling model is “Do today’s work today”, meaning that appointments are made on the same day or within 48 hours^(8,13)^.

However, the lack of consensus around Advanced Access is partly due to important drawbacks. The model pressures clinicians to see unscheduled patients^(14)^, which can lead to an excessive workload^(15,16)^ and staff anxiety^(16)^. This situation is exacerbated by inadequate implementation infrastructure^(15)^, insufficient staff training, and low public awareness of AA^(17)^.

Conversely, many studies report benefits from implementing AA in Primary Health Care, including improved quality of care^(18)^, easier access, and an increased number of appointments^(19)^, as well as reductions in secondary-care visits^(19)^ and in the no-show rate for appointments^(6)^.

A scoping review^(20)^ identified a self-reflective tool for healthcare professionals using AA, titled *Outil Réflexif sur l’Accès Adapté -* ORAA *professionnel* (ORAAprof), developed by Canadian researchers. The tool enables health professionals to assess the level of implementation and identify recommendations to improve AA^(21)^. However, despite these benefits, we found no published translations or cross-cultural adaptations of the instrument.

In Brazil, AA has been implemented in some health services-mainly in Primary Health Care^(6,19,20,22,23)^-but there is no instrument available for analysis, assessment, or professional self-reflection.

Given this gap, a tool is needed to support professionals’ self-reflection within Brazilian health services, aiming to contribute to improvements in Advanced Access within the Unified Health System (SUS). Therefore, this context justifies the translation and cross-cultural adaptation of *Outil Réflexif sur l’Accès Adapté* (ORAAprof) into Brazilian Portuguese.

## OBJECTIVES

To translate and cross-culturally adapt the self-reflective tool *Outil Réflexif sur l’Accès Adapté* (ORAAprof) into Brazilian Portuguese.

## METHODS

### Ethical considerations

The study protocol was approved by the Research Ethics Committee of the Municipal Health Secretariat of São Paulo (SMS/SP) and by the Research Ethics Committee of the School of Nursing, University of São Paulo, in accordance with Brazilian National Health Council Resolution No. 466/2012. Authorization to translate and cross-culturally adapt *Outil Réflexif sur l’Accès Adapté* (ORAAprof) into Brazilian Portuguese was obtained from the original instrument authors via email. The Informed Consent Form (ICF) was obtained from all study participants electronically.

This research is part of the umbrella project “Implementation of Advanced Access in Health Units: Processes and Outcomes”, funded by the National Council for Scientific and Technological Development (CNPq), Directorate of Agricultural, Biological and Health Sciences (DCABS), with a focus on innovation research. This article forms part of the doctoral thesis entitled “Advanced Access: a possibility to reorganize access management in the Unified Health System”, submitted to the Graduate Program in Nursing, School of Nursing, University of São Paulo (2023).

### Study type

This methodological study aimed to translate and cross-culturally adapt *Outil Réflexif sur l’Accès Adapté - ORAA professionnel* (ORAAprof)^(21)^ from French into Brazilian Portuguese. Methodological studies are designed to organize and analyze data that support the translation and adaptation of instruments^(24)^, following a set of ordered procedures to ensure equivalence between the source and target versions^(25,26)^.

### Methodological procedures

**Figure 1:**
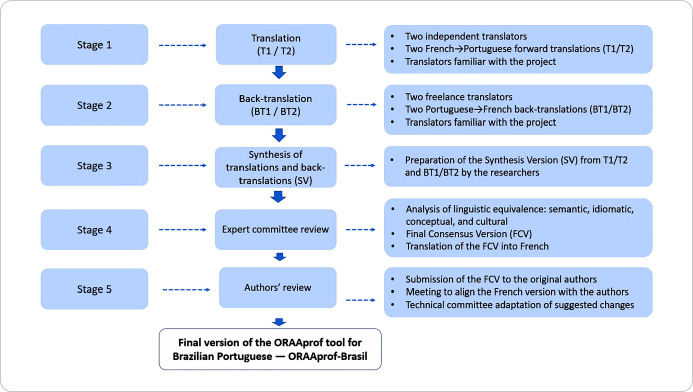
Stages of the translation and cross-cultural adaptation of ORAA *professionnel* into Brazilian Portuguese

To achieve the proposed objective, we followed Guillemin et al.’s recommendations^(25)^, adapted to this study context, in five stages: (1) translation, (2) back-translation, (3) synthesis version, (4) expert committee review, and (5) approval by the original instrument authors (Figure 1).

### Study setting and data collection

The data-collection settings varied according to each stage of the research. As shown in Figure 1, the first stage of the translation process from French (T1 and T2) into Brazilian Portuguese was carried out by two independent translators who were native speakers of Brazilian Portuguese, fluent in French, affiliated with linguistics, and without healthcare backgrounds.

In the second stage, back-translations from Brazilian Portuguese into French (BT1 and BT2) were independently performed by two linguistics professionals proficient in French and without clinical experience.

In the third stage, the researchers selected the best-expressed phrases and produced the synthesis version (SV) by reconciling T1, T2, BT1, and BT2.

In the fourth stage, the SV (translations and back-translations) and the original French version were submitted to an expert committee of five judges for evaluation, as recommended by Ayre and Scally^(27)^. The committee was selected through convenience sampling and is formed by five bilingual judges (Portuguese-French): two nurses, one physician, one social scientist, and one Portuguese language lecturer.

The multidisciplinary expert committee evaluated the qualitative aspects of the translation and adaptation of ORAAprof using linguistic-equivalence criteria at the semantic, idiomatic, cultural, and conceptual levels. For this purpose, an assessment form was developed containing instructions, the full content of *Outil Réflexif sur l’Accès Adapté - ORAA professionnel*, and fields for rating linguistic equivalence (Appendix 1). Invitations were sent by email. After acceptance, individual online meetings were held to brief the judges on the study and to obtain their electronically signed ICF (see Appendix 2). The researchers remained available to answer any queries.

In the fifth stage, the final consensus version (FCV) was back-translated into French by an independent linguistics professional. That version was sent to the original authors for review and approval. Following their approval, the final Brazilian Portuguese version of ORAAprof was produced, titled ORAA - *Ferramenta Autorreflexiva sobre o Acesso Avançado para Profissionais* (ORAAprof-Brasil) - translated as “Self-Reflective Tool for Professionals on Advanced Access” and hereafter, when used in its abbreviated form, referred to as ORAAprof-Brazil.

### Data analysis

The qualitative analysis of the instrument assessed semantic equivalence, which verifies whether word meanings were translated correctly (vocabulary and grammar), given that a term may assume different meanings across cultures. Idiomatic equivalence concerns the translation of idioms or colloquialisms that, being tied to the source culture, may be difficult to render literally; in such cases, translators should formulate an equivalent expression that makes sense in the target language. Cultural equivalence involves adapting items to reflect experiences typical of the target culture; translators should substitute items with appropriate equivalents that express the lived experiences of that culture. Finally, conceptual equivalence verifies whether translated terms retain the same conceptual value and thus preserve the original construct^(26)^.

The quantitative analysis employed the item-level Content Validity Ratio (I-CVR) and the scale level Content Validity Ratio (S-CVR), both calculated using Lawshe’s method to minimize the likelihood of chance agreement^(27)^. Expert committee members were instructed to score each item as “1” if they agreed the item was equivalent (for each domain: semantic, idiomatic, cultural, and conceptual) and as “0” if they disagreed, following Equation 1 ^(28)^.

**Equation 1: Content Validity Ratio (CVR).**


$$CVR=\frac{\mathrm{ne}-N/2}{N/2}$$

*ne - number of expert committee members who indicate agreement, N - total number of committee members.*

Source: Lawshe, 1975.

According to Ayre & Scally, for an expert committee of five members, both the item-level and scale-level CVR must equal 1.0. In cases of disagreement, researchers revise the item(s) based on the suggestions, implement the modifications, and resubmit the material to the committee for re evaluation^(27)^. The outcome of this stage produced the final consensus version (FCV).

## RESULTS

The original *ORAAprof* instrument, the forward translations (T1 and T2), and their respective back-translations (BT1 and BT2) were reviewed by the research team, which prepared the synthesis version (SV) (see Appendix 3). After submission to the expert committee for evaluation and approval, the SV produced the FCV. The FCV was then submitted to the original authors; their approval resulted in the final version.

For some items, T1 was prioritized over T2. The translators’ renderings for Items 3-8 (initial instructions) were highly similar, almost identical.

For Item 1 - Title, *Outil Réflexif sur l’Accès Adapté*, T1’s rendering, ORAA *- Ferramenta Reflexiva sobre o Acesso Adaptado*, was selected because it conveyed a meaning closer to the original. For Item 2, regarding the phrase “*qui permet aux médecins et autres professionnels de la santé de première ligne*”, T1 (“*que permite a médicos e outros profissionais de saúde da linha de frente*”) better reflected the intended sense than T2 (“*que permite aos médicos e demais profissionais de saúde de primeira linha*”), since “*primeira linha*” could be mistakenly interpreted as referring to the interviewed professional’s quality rather than their service position.

Throughout the questionnaire, the term “*questão*” (T2 version) was used instead of “*pergunta*” (T1 version). For items concerning the questionnaire content, the translations differed mainly in verb choice while preserving the original meaning of the items.

The qualitative analysis of ORAAprof was conducted by the multidisciplinary expert committee of five members through assessment of semantic, idiomatic, conceptual, and cultural equivalence. The quantitative analysis employed the item-level Content Validity Ratio (I-CVR) and the scale-level Content Validity Ratio (S-CVR). The validation process proceeded in three stages: (1) instrument evaluation by the expert committee; (2) item revision by the researchers based on the committee’s qualitative and quantitative feedback; and (3) committee re-evaluation of the revised instrument. The entire process comprised three rounds. In all rounds, expert committee members had access to the synthesis version, the back-translations, and the original French version of the instrument.

In the first evaluation round conducted by the expert committee, of the 42 items assessed, 30 presented disagreement (I-CVR < 1.0). These items were justified by the committee members, revised by the researchers according to the suggestions, and resubmitted to the committee. In that round, the scale-level Content Validity Ratio (S-CVR) was 0.86.

The first round was the longest stage of the study, lasting six months. The research team experienced difficulties recruiting healthcare professionals fluent in French who were available to participate. After this barrier was overcome and the committee reached its target of five members, one participant withdrew, stating they could not contribute to the study; it took approximately 30 days to recruit a replacement. Two semantic errors introduced during transcription of the SV were subsequently identified and corrected. In addition, the return of evaluations from two committee members was delayed due to scheduling conflicts, which further extended completion of the round.

In the second round, prior to resubmission to the committee, the researchers replaced the term “*Acesso Adaptado*” with “*Acesso Avançado*” throughout the instrument, following the original *I’Accès Adapté*, because “Acesso Avançado (Advanced Access)” is the internationally recognized label for the scheduling model. Of the 30 items re-evaluated, four did not reach the target CVR of 1.0; the committee justified these items. The committee reported that three of the four had semantic issues and one had a conceptual issue. The terminology change was accepted unanimously. In that round, the S-CVR was 0.99. The researchers reviewed the suggestions and implemented the corrections.

In the third round, after an extensive review, the research team decided to use the term “*paciente*” (patient) when the instrument referred to personal clinical relationships (e.g., “*meu paciente*(*s*)”) and “*usuário do serviço de saúde*” (service user) when reference was to the service. In this round, all items achieved the target CVR; the S-CVR, therefore, equalled 1.0. Regarding this terminology change, all committee members agreed; Judge 4 commented: “It is coherent and culturally more acceptable for what we believe - for the way people use the health service - to substitute ‘patient’ with ‘user’”.

The translation and adaptation process - comprising the qualitative assessment of linguistic equivalence conducted by the expert committee, the quantitative evaluation using the item-level Content Validity Ratio and the scale-level Content Validity Ratio, and iterative revisions by the research team - resulted in the consensus translation titled ORAA *Profissional - Ferramenta Autorreflexiva sobre o Acesso Avançado para Profissionais* (ORAAprof-Brasil) (see Appendix 4).

## DISCUSSION

Translations and cross-cultural adaptations of questionnaires into other languages have increased^(29)^. However, to date, we are not aware of any translation or adaptation of ORAAprof for use in another country. Thus, this study is both novel and relevant to the Brazilian context, as it successfully achieved the proposed cross-cultural adaptation and may contribute to improving timely access within the SUS.

Using an instrument in a new linguistic setting requires careful analysis grounded in the cultural context to ensure reliability when accounting for linguistic and cultural differences^(30)^. Consequently, specific methods are needed to achieve equivalence between the source and target languages, alongside content validation^(26)^. To meet this aim, the present study followed established methodological guidelines^(25)^ to minimize bias and avoid misleading results.

Therefore, ORAAprof-Brazil is suitable for use by health professionals as a self-reflective instrument to evaluate Advanced Access and help improve timely access. Employing a self administered instrument reduces costs and time for research development and enables comparisons with international studies^(31)^.

The translation (stage 1) and back-translation (stage 2) were conducted in a literal and formal manner. In stage 3, the researchers proceeded with the chosen method to produce the SV.

The expert committee’s analysis of linguistic equivalence provided an appropriate framework for adapting the instrument to Brazilian Portuguese. The qualitative analysis ensured that the final version was understandable and applicable in the local context. For this purpose, the expert committee based its assessment on semantic, idiomatic, cultural, and conceptual equivalence^(25,26)^.

Thus, our findings align with the cited literature: the expert committee conducted three thorough rounds of linguistic evaluation to secure semantic equivalence (word meaning), idiomatic equivalence (expressions characteristic of the target language), cultural equivalence (terms appropriate to the Brazilian context), and conceptual equivalence (preservation of the original construct).

The study findings reflect the research team’s careful attention during this stage, particularly when they proposed replacing the term “*paciente*” with “*usuário do serviço de saúde*”. The rationale was that *paciente* is commonly associated with illness, while not all individuals who seek services are ill, even though they may all present a health-related need. The research team retained *patient* for items with a personal clinical meaning and used *service user* (Portuguese: “*usuário*” or “*usuário do serviço de saúde*”) for items referring to the service. This cultural adaptation is supported by the etymology of “*paciente*” (relating to patience) and by the patient-professional relationship, which implies closeness; by contrast, “*usuário*” evokes people who make use of a service^(32)^.

Quantitative measures were calculated using the item-level Content Validity Ratio (I-CVR) and the scale-level Content Validity Ratio (S-CVR), which, according to Lawshe (1975), support content validity and reduce the likelihood that disagreements occurred by chance. The results show that in the first round, the S-CVR was 0.86 (agreement on 12 of 42 items); in the second round, the S CVR reached 0.99 (agreement on 38 items); and in the final round, the S-CVR equalled 1.0 (agreement on all items of the instrument).

This study enabled the translation and cross-cultural adaptation of the self-reflective instrument for health professionals - ORAA *Profissional - Ferramenta Autorreflexiva sobre o Acesso Avançado para Profissionais* (ORAAprof-Brasil) - for use in Brazil, based on the French questionnaire *Outil Réflexif sur l’Accès Adapté -* ORAA *professionnel* ^(13)^. The self-reflection fostered by ORAAprof encourages professionals to consider access guided by the five contemporary pillars of Advanced Access: (1) comprehensive planning that accounts for patients’ needs, service provision, and recurring variations; (2) regular adjustment of supply and demand; (3) an appointment scheduling system; (4) integration and optimization of collaborative practice; and (5) communication and Advanced Access functionalities^(33)^.

Recognizing the complexity of the SUS, critiques of the Advanced Access model, and macro level limitations (federal, state, and municipal policy and governance), ORAAprof-Brazil offers a practical roadmap for self-reflection and local adaptation by health professionals at the micro level (the local service and the team’s or clinician’s schedule).

By addressing topics ranging from demand-and-supply planning to interprofessional communication and service-user satisfaction^(33)^, the tool promotes a critical appraisal of access to appointments and services, prompting professionals to seek feasible solutions within identified constraints and challenges.

Reflection on the applicability of each item in the context of a clinician’s personal schedule can lead to adjustments and reprioritizations that render access more effective and equitable, since the tool takes into account the specific needs of the population in the professional’s territory and the reality of available resources.

### Study limitations

This study rigorously followed the prescribed methodological steps; however, several limitations should be considered. First, we did not perform a pretest to evaluate the instrument’s suitability with respect to comprehension difficulties^(34)^.

A second limitation relates to instrument validation: although beyond the scope of the present study, psychometric validation is the stage that provides greater evidence of validity and reliability.

A final limitation is the absence of a reliability analysis; this gap presents a clear avenue for continuing this research in future studies.

### Contributions to nursing, public policy, and access management

This study provides a valuable instrument for nursing, public policy, and access management in the SUS by making available a self-reflective questionnaire/tool on access management with a focus on Advanced Access, translated and adapted into Brazilian Portuguese. This initiative will enable professionals and managers to monitor and evaluate access management and to implement evidence-based changes to promote timely access in Primary Health Care.

## FINAL CONSIDERATIONS

This study describes the translation and cross-cultural adaptation process through linguistic equivalence analysis and approval by the original authors-a fundamental phase in adapting an instrument to another language. The final Brazilian Portuguese version of the questionnaire, ORAA *Profissional - Ferramenta Autorreflexiva sobre o Acesso Avançado para Profissionais* (ORAAprof-Brasil), preserves linguistic equivalence with the original instrument.

ORAAprof-Brazil enables health professionals to reflect on how Advanced Access operates and on the essentials for informed decision-making and model refinement. Moreover, because it is self administered, its use reduces costs and time in research and facilitates comparison with international studies.

ORAAprof is an up-to-date instrument grounded in the five contemporary pillars of Advanced Access. Considering service users’ needs and the dynamic management of supply and demand, it supports action planning, schedule review, promotion of interprofessional care, and continuous improvement of communication and model functioning.

It is important to emphasize the need to further validate ORAAprof-Brazil, encompassing both validity and reliability assessments.

## Data Availability

The research data are available in a repository: https://doi.org/10.7910/DVN/DNE9ZT
