# Supplementary material for: Translation and cross-cultural adaptation into Brazilian Portuguese of the ORAAprof instrument for Advanced Access
Source: Rev Bras Enferm. 2025 Dec 8;78(Suppl 3):e20240329. doi: 10.1590/0034-7167-2024-0329 (PMC12707777; doi:10.1590/0034-7167-2024-0329)
Supplement: Translation

# Translation: Tradução e adaptação transcultural para o português/Brasil do instrumento ORAAprof para o Acesso Avançado

**DOI:** 10.1590/0034-7167-2024-0329pt

**Authors:** Alexandre Ramiro Pinto, Lislaine Aparecida Fracolli, Lucia Yasuko Izumi Nichiata

**Affiliations:** I Universidade de São Paulo. São Paulo. São Paulo. Brasil

**Keywords:** Tradução, Métodos, Agendamento de Consultas, Acessibilidade aos Serviços de Saúde, Atenção Primária à, Saúde.

## Abstract

**Objetivos:**

traduzir e adaptar transculturamente a ferramenta autorreflexiva *Outil Réflexif sur I’Accès Adapté* (ORAAprof) para o português do Brasil.

**Métodos:**

pesquisa metodológica desenvolvida de 08/2022 a 04/2023 em cinco etapas: tradução; retrotradução; síntese das traduções; avaliação por um comitê juízes; e aprovação dos autores.

**Resultados:**

as etapas de traduções e retrotraduções foram realizadas por especialistas independentes; a elaboração da versão-síntese baseada nas traduções e retrotraduções foi efetuada pelos pesquisadores brasileiros; a avaliação da versão-síntese conduzida pelo comitê de juízes considerou a análise linguística (semântica, idiomática, cultural e conceitual) e julgou como adequada a versão final para o português, com Razão de Validade de Conteúdo Geral igual a 1,0; a versão consensual da Ferramenta Autorreflexiva sobre o Acesso Avançado para Profissionais (ORAAprof-Brasil) foi aprovada pelos autores originais.

**Considerações Finais:**

o ORAAprof-Brasil pode fornecer informações para melhorar a qualidade e a gestão do acesso na Atenção Primária em Saúde.

## INTRODUÇÃO

De acordo com a Organização Mundial da Saúde, a qualidade do atendimento dos serviços de saúde aumenta os resultados de saúde desejados. Para isso, é esperado que estes ofereçam cuidados baseados em evidências (eficazes), evitem danos (seguros) e realizem a assistência para atender às necessidades dos usuários (centrados na pessoa). Dessa maneira, um ponto crucial é que o acesso deve ser oportuno, com foco na redução do tempo de espera e dos atrasos^(1)^. O acesso oportuno também é entendido como a capacidade do serviço de saúde de agendar ou marcar uma consulta o mais rapidamente possível para responder à necessidade trazida pelo usuário. Argumenta-se que o agendamento organizado diminui os custos e a superlotação em outros níveis de atenção^(2)^.

O acesso oportuno aos serviços de saúde é um dos maiores desafios dos sistemas de saúde no contexto internacional^(3)^, sendo considerado a primeira barreira a ser superada pelo usuário do serviço de saúde^(4)^. No Brasil, existem outras barreiras que dificultam ainda mais o acesso ao Sistema Único de Saúde (SUS), como o subfinanciamento, o elevado número de usuários por equipe da Estratégia Saúde da Família (ESF), a escassez e má distribuição de médicos, problemas operacionais e subdimensionamento dos serviços, incluindo formalidade excessiva no agendamento de consultas (distribuição de senha e limitação de horários) e priorização seletiva de grupos^(5,6)^.

A melhoria do acesso requer estratégias de organização que considerem a integração e equilíbrio entre oferta e demanda^(7)^, como conhecer o tamanho da população cadastrada (tamanho do painel) e o número de consultas disponíveis (agenda); rejeitar a classificação de “urgente” versus “não urgente”; e identificar as necessidades da procura por consultas^(8)^. Alguns modelos de agendamento de consultas têm sido implementados para alcançar esse equilíbrio, como o Acesso Aberto, *Book on the Day, Supesaturate, Carve-out* e Acesso Avançado^(9)^.

O Acesso Avançado (AA) foi desenvolvido em 1998, nos Estados Unidos, pelo médico Mark Murray e pela enfermeira Catherine Tantau^(10)^, com posterior expansão para a América do Norte e Europa^(11,12)^. No AA, o usuário do serviço de saúde deve ser atendido em até 48 horas, e não há distinção entre os tipos de consultas de urgência (demanda espontânea) ou de rotina (demanda programada). O slogan desse modelo de agendamento é “Faça todo o trabalho de hoje, hoje”, de modo que o agendamento é realizado no mesmo dia ou em até 48 horas^(8,13)^.

Entretanto, a falta de unanimidade na aceitação do Acesso Avançado deve-se, em parte, a problemas significativos. O modelo pressiona os profissionais a atenderem pacientes não agendados^(14)^, o que, por sua vez, causa sobrecarga inadequada^(15,16)^ e ansiedade na equipe^(16)^. Essa situação é agravada por fatores como infraestrutura inadequada para a implantação^(15)^, falta de capacitação da equipe e desconhecimento da população sobre o AA^(17)^.

Por outro lado, há muitos estudos que apontam benefícios da implantação desse modelo na Atenção Primária à Saúde, tais como: melhora na qualidade da atenção^(18)^; facilitação do acesso, aumento do número de consultas^(19)^, bem como diminuição dos atendimentos na atenção secundária^(19)^ e da taxa de não comparecimento nas consultas^(6)^.

Em uma revisão de escopo^(20)^, foi identificada uma ferramenta autorreflexiva para profissionais de saúde que utilizam o AA, intitulada *Outil Réflexif sur I’Accès Adapté - ORAA professionnel* (ORAAprof), desenvolvida por pesquisadores canadenses. Ela possibilita aos profissionais da saúde avaliarem o nível de implementação e identificar recomendações para melhorar os AAs^(21)^. No entanto, apesar dos benefícios, ainda não encontramos sua tradução e adaptação para outros países.

No Brasil, há experiências de implantação do AA nos serviços de saúde, principalmente na Atenção Primária^(6,19,20,22,23)^, mas não há um instrumento de análise/avaliação/autorreflexão para os profissionais da saúde.

Diante dessa ausência, surge a necessidade de uma ferramenta que auxilie a autorreflexão dos profissionais na realidade dos serviços de saúde brasileiros, visando contribuir para a melhoria do Acesso Avançado no Sistema Único de Saúde (SUS). Portanto, esse contexto justifica a realização da tradução e adaptação transcultural do ORAAprof para o português do Brasil.

## OBJETIVOS

Traduzir e adaptar transculturamente a ferramenta autorreflexiva *Outil Réflexif sur I’Accès Adapté* (ORAAprof) para o português do Brasil.

## MÉTODOS

### Aspectos éticos

O desenho do estudo foi aprovado pelo Comitê de Ética e Pesquisa (CEP) da Secretaria Municipal de Saúde de São Paulo (SMS/SP) e pelo CEP da Escola de Enfermagem da Universidade de São Paulo, atendendo à Resolução 466/2012. O processo de tradução e adaptação transcultural do ORAAprof para o português do Brasil foi autorizado pelos autores do instrumento original via e-mail. O Termo de Consentimento Livre e Esclarecido (TCLE) foi obtido de todos os indivíduos envolvidos no estudo por meio digital.

Esta pesquisa é parte integrante do projeto guarda-chuva “Implementação do Acesso Avançado em Unidades de Saúde: processos e resultados”, financiado pelo Conselho Nacional de Desenvolvimento Científico e Tecnológico (CNPq) da Diretoria de Ciências Agrárias, Biológicas e de Saúde (DCABS), com enfoque em pesquisas de inovação. Este artigo é parte da tese de doutorado apresentada ao Programa de Pós-Graduação em Enfermagem da Escola de Enfermagem da Universidade de São Paulo, com o título “Acesso Avançado: uma possibilidade para reorganizar a gestão do Acesso no Sistema Único de Saúde”, publicada em 2023.

### Tipo de estudo

Trata-se de um estudo de caráter metodológico que propõe a tradução e adaptação transcultural do *Outil Réflexif sur I’Accès Adapté - ORAA professionnel* (ORAAprof)^(21)^ do francês para o português do Brasil. O estudo metodológico caracteriza-se pela organização e análise de dados para auxiliar na tradução e adaptação de instrumentos^(24)^, por seguir um conjunto ordenado de procedimentos que garantem a equivalência entre a ferramenta no idioma original e o de destino^(25,26)^.

### Procedimentos metodológicos

Para alcançar o objetivo proposto, foram consideradas as recomendações de Guillemin e colaboradores^(25)^, adaptando-as ao cenário desta pesquisa em cinco etapas: 1) tradução, 2) retrotradução, 3) versão-síntese, 4) avaliação por comitê de juízes, 5) aprovação por parte dos autores originais do instrumento, conforme a Figura 1.

**Figura 1:** Estágios da tradução e adaptação transcultural do “*ORAA professionnel*” para o português falado no Brasil

### Cenário do estudo e coleta de dados

O cenário de coleta dos dados foi diversificado de acordo com cada etapa da pesquisa. Como apresentado na Figura 1, a primeira etapa do processo de tradução do francês (T1 e T2) para o português do Brasil foi realizada por dois tradutores independentes, falantes nativos do português do Brasil e fluentes no francês, ligados à área da linguística e leigos na área da saúde.

Na segunda etapa, foi efetuada a retrotradução do português do Brasil para francês (RT1 e RT2), também de forma independente por dois tradutores da área da linguística, proficientes em francês e leigos na área da saúde.

Na terceira etapa, os pesquisadores selecionaram as frases de melhor expressão e elaboraram a versão-síntese (VS) com base na mescla das versões T1 e T2, RT1 e RT2.

Na quarta etapa, a VS (traduções e retrotraduções) e a versão original em francês foram enviadas para avaliação por um comitê formado por cinco juízes, conforme indicado por Ayre e Scally^(27)^. Esse comitê foi escolhido por conveniência e composto por cinco juízes bilíngues (português e francês): dois enfermeiros, um médico, um cientista social e um docente de língua portuguesa.

Esse comitê multiprofissional foi formado para avaliar o conteúdo qualitativo de tradução e adaptação do ORAAprof por meio da equivalência linguística de ordem semântica, idiomática, cultural e conceitual. Para isso, foi desenvolvido um instrumento com as orientações, todos os elementos do *Outil Réflexif sur I’Accès Adapté - ORAA professionnel* e os campos para análise da equivalência linguística (Apêndice 1). Foi feito o convite por e-mail; após a aceitação, foi agendada uma reunião on-line. Em seguida, foi realizada a reunião on-line de forma individual para esclarecer os juízes sobre a pesquisa e solicitar a assinatura digital do TCLE (Apêndice 2). Os pesquisadores ficaram à disposição dos juízes para sanar qualquer dúvida.

Na quinta etapa, a VCT foi traduzida para o francês por um profissional da área da linguística, independente. Essa versão foi enviada para análise e aprovação dos autores. Após aprovação, foi produzida a versão final do ORAAprof para o português do Brasil, intitulada “ORAA - Ferramenta Autorreflexiva sobre o Acesso Avançado para Profissionais (ORAAprof-Brasil)”.

### Análise dos dados

A análise qualitativa do instrumento verificou a equivalência semântica, que consiste em certificar se houve tradução correta dos significados das palavras (vocabulário e gramática), visto que uma palavra pode assumir um significado diferente a depender da cultura à qual está submetida. A equivalência idiomática refere-se à tradução de expressões idiomáticas ou coloquialismos que, por estarem ligados à cultura de origem, podem ser difíceis de traduzir. Nesses casos, o tradutor deve formular uma expressão equivalente que faça sentido no idioma de destino. Já a equivalência cultural corresponde ao processo de obter os itens experimentados em determinada cultura. Assim, os tradutores buscarão realizar a substituição do item por outro equivalente que expresse as experiências de vida daquela cultura. Por fim, a equivalência conceitual é a etapa que verifica se as palavras traduzidas têm o mesmo valor conceitual, preservando o conceito original^(26)^.

A análise quantitativa foi realizada por meio da Razão de Validade de Conteúdo Individual (CVR-I) e Geral (CVR-G), ambos calculados por meio do método de Lawshe, visando evitar possíveis concordâncias ao acaso^(27)^. Para o cálculo, os juízes foram orientados a pontuar o item com “1” se concordassem com a equivalência para cada item (de cada esfera: semântica, idiomática, cultural e conceitual) e com “0” se discordassem, conforme a equação descrita na Equação 1^(28)^.

**Equação 1: Razão de Validade de Conteúdo (CVR - *Content Validity Ratio*).**


$$CVR=\frac{\mathrm{ne}-N/2}{N/2}$$

*ne - número juízes que concordam, N - número total de juízes participantes.*

Fonte: Lawshe, 1975.

De acordo com Ayre & Scally, quando o comitê de juízes é formado por cinco especialistas, o CVR individual e o geral devem ser iguais a 1,0. Nos casos em que houver discordância, os pesquisadores revisam o(s) item(ns) com base na(s) sugestão(ões), realizam a(s) modificação(ões) e retornam o material ao comitê para nova análise^(27)^. O produto desse estágio conduziu a versão consensual da tradução (VCT).

## RESULTADOS

O instrumento original do ORAAprof, as traduções (T1 e T2) e suas respectivas retrotraduções (RTI e RT2) foram analisadas pelos pesquisadores, que elaboraram a versão-síntese - VS (Apêndice 3). Após ser enviada ao comitê de juízes para análise e aprovação, a VS deu origem à VCT. Em seguida, a VCT foi submetida aos autores originais, cuja aprovação resultou na versão final.

Em alguns itens, a versão T1 teve prioridade sobre a T2. As versões feitas pelos tradutores para os Itens 3, 4, 5, 6, 7, 8 (orientações iniciais) foram muito semelhantes, quase idênticas.

No Item 1 - Título, *Outil Réflexif sur I’Accès Adapté,* foi selecionada a T1 “ORAA - Ferramenta Reflexiva sobre o Acesso Adaptado”, pelo fato de essa tradução ter o sentido mais próximo do original. No Item 2, sobre a expressão “*qui permet aux médecins et autres professionnels de la santé de première ligne*”, a versão T1 (“que permite a médicos e outros profissionais de saúde da linha de frente”) expressou melhor o sentido da frase do que a versão T2 (“que permite aos médicos e demais profissionais de saúde de primeira linha”), pois “primeira linha” poderia ser erroneamente interpretado como “qualidade do profissional” entrevistado.

Em todos os itens do questionário, foi utilizada a palavra “questão” (versão T2) no lugar da palavra “pergunta” (versão T1). Nos itens relacionados às questões, as traduções foram muito semelhantes, com variação somente nos verbos, mas mantendo o sentido da pergunta.

A análise qualitativa do ORAAprof foi realizada pelo comitê multiprofissional de cinco juízes mediante a avaliação semântica, idiomática, conceitual e cultural. Já a análise quantitativa foi efetuada por meio da Razão de Validade de Conteúdo individual e geral. O processo transcorreu em três momentos: 1) avaliação da ferramenta pelos juízes; 2) revisão dos itens pelos pesquisadores com base nas sugestões dos juízes, considerando as análises qualitativa e quantitativa; e 3) reavaliação da ferramenta pelo comitê. Destaca-se que todo esse processo foi realizado em três rodadas. Em todas elas, os juízes tiveram acesso à síntese das traduções, às retrotraduções e à versão original do instrumento.

Na primeira rodada de avaliação realizada pelos juízes, dos 42 itens avaliados, houve discordância em 30 itens (CVR-I < 1). Esses itens foram justificados pelos juízes, revisados pelos pesquisadores de acordo com as sugestões e reenviados para o comitê. Nessa rodada, o CVR geral foi igual a 0,86.

A primeira rodada foi a mais extensa da pesquisa, com duração de seis meses. Os pesquisadores tiveram dificuldades de encontrar profissionais da área da saúde com fluência em francês e disponibilidade para participar do estudo. Após superada essa barreira e o comitê estar completo (cinco juízes), houve a desistência de um dos participantes por entender que não poderia contribuir com a pesquisa. O tempo para encontrar o substituto foi de aproximadamente 30 dias. Outro problema enfrentado foi o de dois erros semânticos durante a digitação na VS. Além disso, houve atraso na devolução da análise de dois juízes devido à falta de horário na agenda pessoal, o que prorrogou a finalização dessa rodada.

Na segunda rodada, antes de enviar para a análise dos juízes, os pesquisadores optaram por utilizar em toda a ferramenta o termo “Acesso Avançado” no lugar de “Acesso Adaptado”, conforme traduzido do original “*I’Accès Adapté*”, pois “Acesso Avançado” é como o modelo de agendamento é conhecido internacionalmente. Dos 30 itens avaliados, 4 não alcançaram o CVR desejável, igual a 1,0, sendo estes justificados pelos juízes. Os juízes apontaram que três desses quatro itens tinham erros semânticos e um item apresentava erro conceitual. A mudança do termo foi aceita por unanimidade pelo comitê. Nessa rodada, o CVR geral foi igual a 0,99. Os pesquisadores analisaram as sugestões e realizaram as correções.

Na terceira rodada de análise, após uma ampla pesquisa, os pesquisadores optaram por utilizar o termo “paciente” por entender que o instrumento ORAAprof carrega um sentido pessoal (“meu paciente(s)”) e “usuário do serviço de saúde’” quando a ferramenta referir-se ao serviço. Nessa rodada, todos os itens alcançaram o CVR desejável. Assim, o CVR geral foi igual a 1,0. Em relação à mudança do termo, todos os juízes concordaram com a sugestão, destacando-se a seguinte fala do Juiz 4: *“*É coerente e culturalmente mais aceitável para o que acreditamos, para o modo que as pessoas utilizam o serviço de saúde, fazer a substituição de *paciente* para *usuário.*”

O processo de tradução e adaptação - composto pela avaliação qualitativa de equivalência linguística realizada pelos juízes, avaliação quantitativa calculada por meio da Razão de Validade de Conteúdo individual e geral e revisão pelos pesquisadores - resultou na versão consensual da tradução, denominada “ORAA Profissional - Ferramenta Autorreflexiva sobre o Acesso Avançado para Profissionais (ORAAprof-Brasil)” (Apêndice 4).

## DISCUSSÃO

Traduções e adaptações de questionários para outros idiomas têm crescido^(29)^. Todavia, até o momento, desconhecemos alguma tradução e adaptação do ORAAprof para outro país. Sendo assim, nosso estudo é tanto inédito quanto relevante para o contexto brasileiro, pois alcançou o objetivo proposto de adaptação transcultural, o que contribui para melhorar o acesso ao SUS.

O uso de um instrumento/ferramenta em um novo cenário linguístico exige uma análise minuciosa baseada no contexto cultural, visando garantir a confiabilidade ao considerar as diferenças linguísticas e culturais^(30)^. Por isso, requer o emprego de métodos específicos a fim de alcançar as equivalências entre o idioma de origem e o de destino, em paralelo com a necessidade de validar o conteúdo^(26)^. Para alcançar o objetivo, este estudo adotou as diretrizes metodológicas propostas na literatura^(25)^, a fim de minimizar a ocorrência de viés e resultados tendenciosos.

Portanto, ORAAprof-Brasil está apto a ser utilizado pelos profissionais de saúde como ferramenta autorreflexiva para analisar o AA e melhorar o acesso oportuno. Usar uma ferramenta de autopreenchimento reduz custo e tempo no desenvolvimento de pesquisas e permite a comparação dos resultados com estudos internacionais^(31)^.

Os estágios de tradução (1) e retrotradução (2) aconteceram de modo literal e formal. No terceiro estágio, os pesquisadores deram continuidade no método adotado para garantir a VS.

A análise de equivalência linguística da ferramenta realizada pelo comitê de juízes criou um contexto adequado para sua adaptação ao português do Brasil. A análise qualitativa garante que a versão final seja compreensível e aplicável no contexto local. Para isso, os juízes basearam-se na equivalência semântica, idiomática, cultural e conceitual^(25,26)^.

Assim, os resultados concordam com os autores citados, visto que o estágio de avaliação linguística realizado pelo comitê aconteceu em três rodadas minuciosas em busca das equivalências semântica (significado das palavras), idiomática (expressões características do idioma de destino), cultural (palavras e termos do contexto brasileiro) e conceitual (manutenção do conceito original).

Os resultados da pesquisa revelam o cuidado dos pesquisadores durante esse estágio, ao sugerirem a mudança do termo “paciente” por “usuário do serviço de saúde”, por entenderem que a palavra “paciente” está relacionada a doença, mas nem todos os indivíduos que procuram o serviço possuem uma doença, mas todos têm uma necessidade em saúde. Os pesquisadores mantiveram o uso da palavra “paciente” quando a questão tem um sentido pessoal e “usuário ou usuário do serviço de saúde” quando se relaciona ao serviço. Essa adaptação cultural fundamenta-se nas raízes do termo “paciente”, que se refere à pessoa que tem paciência, e na relação paciente-profissional, que permite uma proximidade; já o termo “usuário” remete à ideia das pessoas que utilizam um serviço^(32)^.

As medidas quantitativas foram realizadas por meio do cálculo da Razão de Validade de Conteúdo individual (CVR-item) e geral (CVR-geral), que segundo Lawshe (1975) garantem a validade do conteúdo. Essa medida assegura que as discordâncias não aconteceram por acaso. Os resultados revelam que, na primeira rodada, o CVR-geral foi igual a 0,86 (concordância em 12 itens); na segunda, a razão alcançou 0,99 (concordância em 38 itens); e somente na última rodada, foi igual a 1,0 (concordância em todos os itens da ferramenta).

A realização deste estudo viabilizou a tradução e adaptação transcultural para uso no território brasileiro da ferramenta autorreflexiva destinada a profissionais de saúde *“*ORAA profissional - Ferramenta Autorreflexiva sobre o Acesso Avançado (ORAAprof-Brasil)”, com base no questionário francês *Outil Réflexif sur I’Accès Adapté - ORAA professionnel* ^(13)^. A autorreflexão proposta pelo ORAAprof estimula os profissionais a refletirem sobre o acesso tendo como referência os cinco pilares contemporâneos do AA: 1) planejamento abrangente considerando as necessidades dos pacientes, a prestação dos serviços e as variações recorrentes; 2) ajuste regular da oferta e demanda; 3) sistema de agendamento; 4) integração e otimização da prática colaborativa; e 5) comunicação e funcionalidades do AA^(33)^.

Ao considerar a complexidade do SUS, as críticas ao modelo de Acesso Avançado e as limitações que estão no macrocontexto (políticas governamentais na gestão federal, estadual e municipal), o ORAAprof-Brasil oferece um roteiro valioso para a autorreflexão e adaptação por parte dos profissionais de saúde no microcontexto (serviço local e agenda da equipe ou do profissional).

Ao abordar tópicos que variam desde o planejamento da demanda e oferta de serviços até a comunicação interprofissional e a satisfação do usuário^(33)^, a ferramenta estimula uma análise crítica do acesso às consultas e serviço, cabendo ao profissional buscar soluções viáveis dentro das limitações e desafios identificados.

A reflexão sobre a aplicabilidade de cada questão no contexto da agenda pessoal pode levar a ajustes e priorizações que tornem o acesso mais eficaz e equitativo, pois considera as necessidades específicas da população do seu território e a realidade dos recursos disponíveis.

### Limitações do estudo

Este estudo seguiu rigorosamente as etapas e os procedimentos metodológicos, contudo algumas limitações devem ser consideradas. A primeira consiste na ausência do pré-teste para avaliar a adequação do instrumento no tocante à dificuldade de compreensão^(34)^.

A segunda limitação relaciona-se à validação do instrumento: apesar de não fazer parte do escopo do estudo, é a etapa que garante maior validade e confiabilidade.

Uma última limitação identificada inclui a ausência de análise de confiabilidade. Essa lacuna abre espaço para a continuidade desta pesquisa em estudos futuros.

### Contribuições para a enfermagem, políticas públicas e gestão do acesso

Este estudo oferece um valioso instrumento para a área da enfermagem, políticas públicas e gestão do acesso ao SUS, ao disponibilizar um questionário/ferramenta autorreflexivo(a) sobre a gestão do acesso com foco no Acesso Avançado, com tradução e adaptação para o português do Brasil. Essa iniciativa possibilitará que os profissionais e gestores monitorem e avaliem a gestão do acesso, bem como promovam mudanças baseadas em evidências, a fim de favorecer o acesso oportuno à Atenção Primária à Saúde.

## CONSIDERAÇÕES FINAIS

Este estudo descreve o processo de tradução e adaptação transcultural por meio da análise da equivalência linguística e aprovação por parte dos autores originais - fase fundamental no processo de tradução e adaptação de um instrumento para outro idioma. A versão final em português falado no Brasil do questionário “ORAA profissional - Ferramenta Autorreflexiva sobre o Acesso Avançado (ORAAprof-Brasil)” mantém equivalência linguística com a versão original.

A ferramenta ORAAprof-Brasil permite que os profissionais de saúde reflitam sobre como o Acesso Avançado funciona e sobre o que é fundamental para tomar decisões e aprimorar o modelo. Além disso, por ser de autopreenchimento, seu uso reduz custos e tempo em pesquisas e facilita a comparação de resultados com estudos internacionais.

O ORAAprof é um instrumento atualizado, pois fundamenta-se nos cinco pilares contemporâneos do Acesso Avançado. Considerando as necessidades dos usuários e gerenciamento dinâmico da oferta e demanda, ele permite realizar um planejamento das ações, revisar os agendamentos, promover cuidado interprofissional e melhorar constantemente a comunicação e funcionamento do modelo.

Ressalta-se a importância de validar a ferramenta ORAAprof-Brasil, o que envolve medidas de validade e confiabilidade.

## Data Availability

Os dados de pesquisa estão disponíveis em repositório: https://doi.org/10.7910/DVN/DNE9ZT.
